# Supplementary material for: Soy protein isolate-carboxymethyl cellulose conjugates with pH sensitivity for sustained avermectin release
Source: R Soc Open Sci. 2019 Jul 17;6(7):190685. doi: 10.1098/rsos.190685 (PMC6689608; doi:10.1098/rsos.190685)
Supplement: GPC test results of CMC [file rsos190685supp2.pdf]

Cirrus GPC Sample Injection Report

Analysis by PL GPC 50 | Date Collectd: 2019-1-25 10:47:52

Workbook: E:\Cirrus Workbooks\PEO analysis\PEO analysis.plw

Sample Details | Sample Name: 1901183603

Acquired: 2019-1-25 10:47:52 | Concentration: 1.85 mg/mlInjection Volume: 100.0 ul

Analysis Using Method: PEO PEG | **Calibration Used: 2019-1-18 13:44:32**

Calibration Type: Narrow Standard | Curve Fit Used: 1 | K: 14.1000 | Alpha: 0.7000

Calibration Curve: y = 10.846873 - 0.430877x^1

High Limit MW RT: 12.25 mins | Low Limit MW RT: 19.11 mins

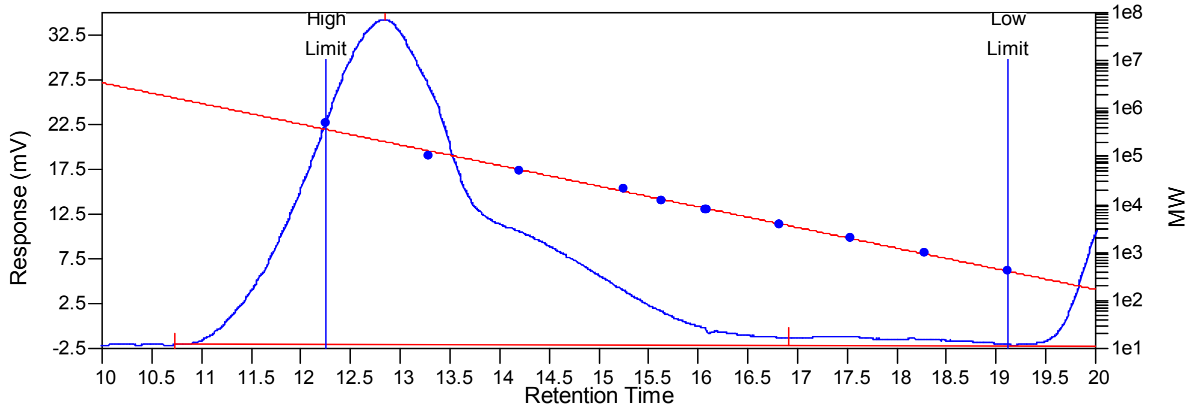

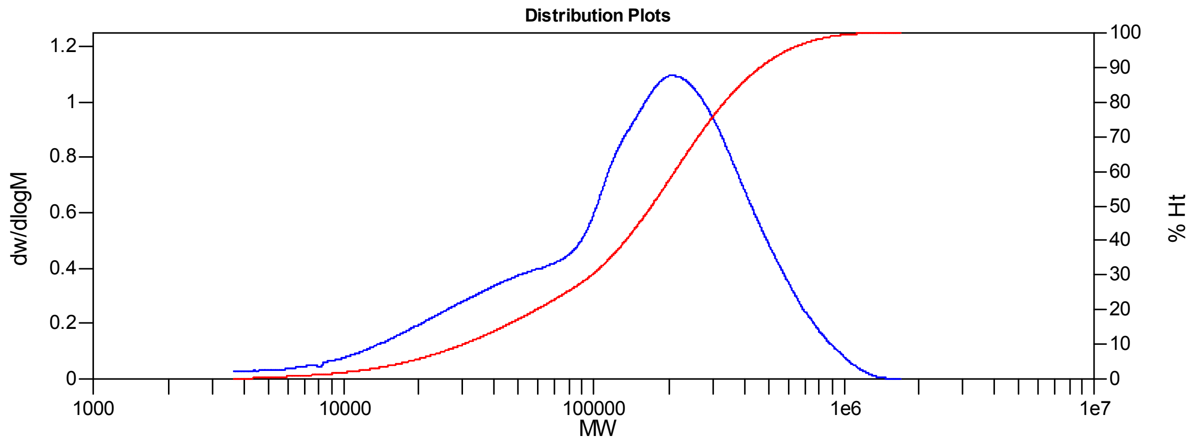

MW Averages

Peak No	Mp	Mn	Mw	Mz	Mz+1	Mv	PD
1	204901	69987	216329	382181	544861	194059	3.09099

Processed Peaks

Peak No	Name	Start RT (mins)	Max RT (mins)	End RT (mins)	Pk Height (mV)	% Height	Area (mV.secs)	% Area
1		10.73	12.85	16.91	36.3139	0	4613.74	100
